# Revisiting Short-term Outcomes of Conventional and Computer-Assisted Total Knee Arthroplasty: A Population-based Study

**DOI:** 10.5435/JAAOSGlobal-D-22-00089

**Published:** 2022-06-10

**Authors:** Abdalrahman G. Ahmed, Yao Tian, Mohamed Hasan, Alexandra Harris, Hassan M. K. Ghomrawi

**Affiliations:** From the Medical College of Wisconsin, Milwaukee, WI (Ahmed); the Department of Surgery (Dr. Tian and Dr. Ghomrawi), Surgical Outcomes and Quality Improvement Center (Dr. Tian), Health Sciences Integrated PhD Program (Dr. Hasan and Harris), Center for Health Services and Outcomes Research (Dr. Ghomrawi), the Department of Medicine (Rheumatology) (Dr. Ghomrawi), the Department of Pediatrics (Dr. Ghomrawi), Feinberg School of Medicine, Northwestern University, Chicago, IL.

## Abstract

**Background:**

Population-based studies showing the advantage of computer-assisted total knee arthroplasty (CATKA) over conventional total knee arthroplasty (TKA) are outdated. More recent institution-based studies with relatively small sample sizes may hinder wider adoption. This cohort-based study aimed to compare postoperative CATKA and TKA in-hospital complications and 90-day all-cause readmissions using 2017-2018 data.

**Methods:**

Patients who underwent a primary unilateral CATKA or TKA were identified in the New York Statewide Planning and Research Cooperative System database. In-hospital complications were defined based on the 2020 Centers for Medicare & Medicaid Services total hip arthroplasty and TKA complications measure. Ninety-day readmissions were identified using unique patient identifiers. Logistic regression with a generalized estimating equation was used to assess associations of computer assistance with in-hospital complications and 90-day all-cause readmissions.

**Results:**

A total of 80,468 TKAs were identified during the study period, of which 7,395 (9.2%) were CATKAs. Significantly fewer complications occurred among patients who had CATKAs compared with conventional TKAs (0.4% of total CATKAs vs 2.6% of total conventional TKAs, *P* < 0.001); patients who had CATKAs had fewer 90-day all-cause readmissions compared with those who underwent TKAs (363 vs 4,169 revisits, *P* < 0.01). Computer assistance was associated with significantly lower odds of in-hospital complications (odds ratio, 0.15, 95% confidence interval, 0.09 to 0.24; *P* < 0.05) but not 90-day all-cause readmissions.

**Conclusion:**

Patients undergoing CATKAs had markedly lower odds of in-hospital complications, compared with patients having TKAs, which has implications for both patient outcomes and hospital reimbursement. These more recent cohort-based findings encourage wider CATKA adoption.

The overall success of total knee arthroplasty (TKA) procedures relies, in large part, on precise implant positioning, which prevents axis malalignment, increases prosthesis durability, and is associated with better knee function.^1-3^ Although revision rates are generally low,^4,5^ a recent study found that the prevalence of knees with outlier implant positioning measurements requiring revision surgery was high, even among high-volume surgeons.^6^ With TKAs becoming increasingly common, improving the precision of implant positioning may be an important factor in improving patient outcomes.

Compared with conventional TKAs, computer-assisted navigation technology (image based and imageless) increases the accuracy of implant positioning and optimizes the function and longevity of the prosthesis. In a recently published overview of systematic reviews, computer-assisted total knee arthroplasties (CATKAs) were associated with more favorable functional, radiological, and patient safety outcomes.^7^ CATKAs have also been associated with a reduced risk of developing pulmonary emboli and lower rates of postoperative cardiac complications and hematomas.^8-10^ Both image-based and imageless CATKAs were found to reduce in-hospital complications and reduce transfusion risk without markedly increasing hospital length of stay and costs.^11^ However, most of these studies were conducted in small institutions where sample sizes were relatively small and, as a result, may not be generalizable to the broader cohort of patients having TKAs. Moreover, the few published cohort-based studies were conducted using *International Classification of Diseases, Ninth Revision* (*ICD-9*) data, which lack the clinical specificity of *ICD-10* and are not reflective of utilization in recent years.^8,9,10,11,12,13^

In a recently published study spanning 2010 to 2017, a modest increase in the adoption of CATKAs was observed, potentially driven by better patient outcomes.^14^ To our knowledge, however, the relationship between CATKAs and patient outcomes in the *ICD-10* era remains understudied. The primary objective of this study was to compare the rates of postoperative in-hospital complications and 90-day all-cause readmissions for patients having CATKAs and conventional TKAs. We hypothesized that CATKAs would be associated with lower postoperative complications and 90-day all-cause readmissions, compared with conventional TKAs.

## Methods

### Study Design and Data Sources

This was a retrospective study using an all-payer discharge database, the New York Statewide Planning and Research Cooperative System (SPARCS), that collects data on patient characteristics, diagnoses, treatments, outpatient services, ambulatory surgery, and emergency department visits from all nonfederal hospitals in New York that are required to send data to the SPARCS database.^15^ In-hospital data in SPARCS include discharges from all short-term acute care hospitals in New York State. We identified patients who underwent a conventional TKA or CATKA between January 1, 2017, and September 30, 2018. After obtaining institutional review board approval, we queried the SPARCS.

Although the state of New York is not nationally representative, it makes up about 6% of the total US cohort with a notable proportion of racial and ethnic minorities. Its cohort resides in small towns, small cities, and a metropolis. A large number of TKAs are performed in the state,^16^ across practically every type of hospital.^16^ The SPARCS data set has also been used previously for examining complication rates in total joint arthroplasty.^17,18^ Thus, there was a precedent for analyzing in-hospital postoperative complications in New York, such that it may provide important insights into postoperative CATKA complications nationally.

### Study Cohort

Patients who were older than 45 years and received a primary unilateral TKA or CATKA (defined using *ICD-10* Procedure Coding System codes) identifiable in the SPARCS data were included in the study. Of those, patients whose data also included codes for computer assistance (*ICD-10* codes 8E0YXBZ, 8E0YXBF, 8E0YXBG, and 8E0YXBH) were identified as CATKA cases. Those who underwent total hip arthroplasty (THA) within the same hospitalization, who had a bilateral TKA or CATKA, or who had missing discharge data and other covariate information were excluded from our analyses.

### Key Outcomes and Covariates

#### Postoperative In-Hospital Complications

Postoperative in-hospital complications were defined based on the 2020 Centers for Medicare & Medicaid Services (CMS) risk-standardized THA/TKA complications measure. This measure is currently implemented in the Medicare Hospital Value-Based Purchasing program and the Comprehensive Care for Joint Replacement Bundled Payment Model.^19^ CMS's THA/TKA complications measure specifications are annually updated in collaboration with statisticians and clinical experts.^1^ Using *ICD-10-Clinical Modification* and *ICD-10* Procedure Coding System codes, this measure identifies complications in the following clinical categories: acute myocardial infarction, mechanical complications, periprosthetic joint infection/wound infection and other wound complications, pneumonia and other respiratory complications, pulmonary embolism, sepsis/septicemia/shock, surgical site bleeding, and other surgical site complications.^20^ The complete list of *ICD-10* codes included in CMS's THA/TKA complications measure is available at https://qualitynet.cms.gov/inpatient/measures/complication/methodology.

#### 90-Day All-Cause Readmissions

The SPARCS database has unique patient-specific identifiers, which permits longitudinal tracking of patient readmissions in New York State within the period of interest. Any readmission within a 90-day period of discharge was recorded and used for analysis.

### Statistical Analysis

Demographic and clinical characteristics were compared between patients who received conventional TKA and CATKA using Student *t*-tests, chi-square tests, or Fisher exact tests, where appropriate. Means with SDs or numbers of patients with their percentages were used to describe normally distributed continuous variables and categorical variables.

To evaluate the association of practice patterns (conventional TKA versus CATKA) with these outcomes, we used logistic regression models with generalized equation estimation to account for the correlation of patients who underwent conventional TKA and CATKA in the same hospital, while adjusting for demographic and clinical factors. The SPARCS database collects demographic and clinical patient data, including age, sex, race/ethnicity, insurance type, and clinical comorbidities.^15^ We adjusted for variables known to be associated with patient outcomes. The primary variable of interest was the use of computer-assisted navigation technology.^8,9,10,11,12,13,14^ Covariates included age^21^ and insurance type,^22^ as well as the Charlson Comorbidity Index, which was applied to clinical comorbidity data to calculate a measure of disease burden.^23-25^ Race/ethnicity was also included as a covariate because of its known effect on patient access and other outcomes.^14,26-28^ Finally, we adjusted for hospital volume, which was defined using quartiles of combined 2-year procedure volume.^29,30^ Odds ratios (ORs) and their 95% confidence intervals (95% CIs) were estimated and reported, and all *P* values were two-sided with statistical significance evaluated at an alpha level of <0.05. Statistical tests and analyses were performed using SAS 9.4 (SAS Institute).

## Results

Over the study period, 80,468 TKAs were performed in New York, of which 7,395 (9.2%) were CATKAs. Bivariate analysis revealed that demographic variables were generally similar across the conventional TKA and CATKA cohorts, although some differences were observed among the race and insurance-type covariates (Table 1). Non-Hispanic Whites made up a greater proportion of both the CATKA and conventional TKA patient cohort (79.5% vs 70.4%, *P* < 0.0001), whereas Blacks (8.7% vs 10.6%, *P* < 0.0001), Hispanics (4.8% vs 8.6%, *P* < 0.0001), and Asian/Pacific Islanders (1.7% vs 3.1%) made up a much smaller proportion of the overall TKA cohort, particularly the CATKA subgroup. Medicare and Commercial coverage were the most common insurance types among both patients who had CATKAs (44.3% and 47.3%, *P* < 0.0001) and conventional TKAs (45.9% and 39.8%, *P* < 0.0001), whereas Medicaid and Workers' Compensation coverage made up a very small proportion of the CATKAs (3.0% and 2.8%, *P* < 0.0001) and conventional TKAs (6.6% and 3.1%, *P* < 0.0001).

**Table 1:** Demographic and Clinical Characteristics of CATKAs and Conventional TKAs in New York 2017-2018

	CATKA (N = 7,395)	Conventional TKA (N = 73,073)	*P* Value
Female sex, n (%)	4,598 (62.18)	46,427 (63.54)	0.0209
Mean age (yrs) (SD)	67.1 (9.2)	66.9 (9.2)	0.1537
Race/ethnicity, n (%)			<0.0001
Non-Hispanic White	5,881 (79.53)	51,437 (70.39)	
Black	645 (8.72)	7,739 (10.59)	
Hispanic	357 (4.83)	6,287 (8.60)	
Asian/Pacific Islander	127 (1.72)	2,231 (3.05)	
Others	280 (3.79)	4,197 (5.74)	
Unknown	105 (1.42)	1,182 (1.62)	
Insurance, n (%)			<0.0001
Medicare	3,274 (44.27)	33,544 (45.90)	
Medicaid	225 (3.04)	4,851 (6.64)	
Commercial	3,494 (47.25)	29,106 (39.83)	
Workers' Compensation	204 (2.76)	2,254 (3.08)	
Others	198 (2.68)	3,318 (4.54)	
Charlson comorbidity score, n (%)			<0.0001
0	6,492 (87.79)	62,416 (85.42)	
1	716 (9.68)	8,148 (11.15)	
2	154 (2.08)	1,866 (2.55)	
3+	33 (0.45)	643 (0.88)	
Hospital volume, n (%)			<0.0001
<69 cases per year	^a^	1,118 (1.53)	
69-270	216 (2.92)	5,930 (8.12)	
270-661	1,567 (21.19)	14,905 (20.40)	
≥661	5,608 (75.84)	51,120 (68.96)	

CATKA = computer-assisted total knee arthroplasty, SPARCS = Statewide Planning and Research Cooperative System, TKA = total knee arthroplasty

a Owing to SPARCS cell size suppression policy, any values less than 11 (but greater than 0) are masked in the table cells.

The most prevalent complication after a CATKA was mechanical complications (0.19%), followed by pneumonia and pulmonary embolisms. In the unadjusted analyses, there were significantly fewer complications after CATKAs compared with conventional TKAs (0.4% vs 2.6%, *P* < 0.0001) (Table 2). Differences in mechanical complications, periprosthetic joint/wound infections, and sepsis were notable between the two cohorts. Table 3 presents the regression results for the in-hospital complications and 90-day all-cause readmission outcomes. Computer assistance was associated with markedly lower odds of in-hospital complications (OR, 0.15, 95% CI, 0.09 to 0.24). Variables associated with higher odds of in-hospital complications after CATKA included Black race (OR, 1.20, 95% CI, 1.04 to 1.38) and the presence of any comorbidity, although having three or more comorbidities had the highest odds of in-hospital complications (OR, 9.73, 95% CI, 7.63 to 12.4). Computer assistance was not associated with lower odds of readmission, although patients who underwent CATKAs had fewer 90-day all-cause readmissions compared with those who had conventional TKAs (364 vs 4,169 revisits, *P* < 0.01). Male sex (OR, 1.29, 95% CI, 1.19 to 1.39), Medicaid insurance (OR, 1.20, 95% CI, 1.01 to 1.43), and having three or more comorbidities (OR, 2.87, 95% CI, 1.94 to 4.26) were all found to be markedly associated with higher odds of 90-day all-cause readmissions after CATKA. High hospital volume, defined as more than 661 TKA procedures (OR, 0.51, 95% CI, 0.39 to 0.68), was markedly associated with lower odds of 90-day all-cause readmissions after CATKA (Table 3).

**Table 2:** Outcome Characteristics of CATKA and Conventional TKA in New York 2017-2018

Outcomes	CATKA	Conventional TKA	*P* Value
Patients having any complication^a^	28 (0.38)	1,919 (2.63)	<0.0001
Perioperative complication (CMS), n (%)			
Acute myocardial infarction	0	24 (0.03)	0.0989
Mechanical complications	14 (0.19)	1,274 (1.74)	<0.0001
Periprosthetic joint infection/wound infection	^c^	407 (0.56)	<0.0001
Pneumonia	^c^	129 (0.18)	0.1024
Pulmonary embolism	^c^	76 (0.10)	0.7027
Sepsis	^c^	109 (0.15)	0.0026
Surgical site bleeding and others	0	31 (0.04)	0.1103
90-Day all-cause readmissions,^b^ n (%)	363 (5.65)	4,169 (6.48)	0.01

CATKA = computer-assisted total knee arthroplasty, CMS = Centers for Medicare & Medicaid Services, SPARCS = Statewide Planning and Research Cooperative System, TKA = total knee arthroplasty

a A patient can have multiple complications.

b Owing to SPARCS cell size suppression policy, any values less than 11 (but greater than 0) are masked in the table cells.

c To obtain accurate 90-day all-cause readmissions, we only included data until September 2018.

**Table 3:** Associations of CATKA With a Likelihood of In-Hospital Complications and 90-Day All-Cause Readmissions

Parameter	In-Hospital Complications Odds Ratio (95% CI)	90-Day All-Cause Readmissions Odds Ratio (95% CI)
Computer assistance (reference group: conventional)	0.15 (0.09-0.24)^a^	0.90 (0.73-1.12)
Age	1.00 (1.00-1.01)	1.01 (1.00-1.02)
Sex (reference group: female)		
Male	1.09 (0.99-1.20)	1.29 (1.19-1.39)^a^
Race/ethnicity (reference group: non-Hispanic White)		
Black	1.20 (1.04-1.38)^a^	1.04 (0.88-1.22)
Hispanic	0.76 (0.57-1.00)	0.97 (0.72-1.31)
Asian/Pacific Islander	0.53 (0.40-0.72)^a^	0.60 (0.44-0.81)^a^
Others/missing race	0.77 (0.63-0.93)^a^	1.00 (0.84-1.19)
Unknown	1.07 (0.69-1.66)	1.00 (0.72-1.40)
Insurance (reference group: Medicare)		
Medicaid	1.12 (0.90-1.39)	1.20 (1.01-1.43)^a^
Commercial	0.93 (0.81-1.08)	0.84 (0.76-0.94)^a^
Workers' Compensation	1.12 (0.87-1.44)	0.66 (0.48-0.91)^a^
Others	0.84 (0.61-1.16)	1.07 (0.78-1.46)
Charlson comorbidity (reference group: no comorbidity)		
1	3.55 (2.99-4.21)^a^	1.58 (1.40-1.77)^a^
2	6.21 (4.76-8.11)^a^	2.11 (1.70-2.63)^a^
3+	9.73 (7.63-12.4)^a^	2.87 (1.94-4.26)^a^
Hospital volume (reference group: <68.5)		
69-270	0.55 (0.30-1.00)	0.65 (0.48-0.89)^a^
270-661	0.54 (0.31-0.95)^a^	0.60 (0.46-0.79)^a^
≥661	0.72 (0.41-1.25)	0.51 (0.39-0.68)^a^

CATKA = computer-assisted total knee arthroplasty, CI = confidence interval

a *P* < 0.05.

## Discussion

Using a New York all-payer cohort-based data set, this study compared in-hospital complications and 90-day all-cause readmissions among patients aged 45 years and older who underwent either a CATKA or a conventional TKA. We found that patients who underwent CATKAs had notably fewer in-hospital complications. Mechanical complications, periprosthetic joint infections/wound infections, and pneumonia were the most common. After adjusting for covariates, CATKA patients had 85% lower odds of in-hospital complications, compared with those who had conventional TKAs. However, there was no notable association between CATKA and 90-day all-cause readmissions. To our knowledge, this is the first study to analyze complication rates of both conventional TKAs and CATKAs in recent years.

The success of TKAs is rooted in accurate implant positioning to prevent axis malalignment and optimize prosthesis durability while also preventing wear damage and aseptic loosening. Although optimal position for implants is still debatable, CATKA aims to deliver accurate implant positioning and may provide other benefits.^2,3^ Our findings have shown that patients who had CATKAs suffered fewer complications across several categories, which is consistent with older *ICD-9* studies.^4,8,9,10,11,12,13^ We have shown that CATKA patients suffered fewer mechanical complications, compared with conventional TKAs. Our results also showed that patients who had CATKAs had fewer pulmonary embolisms, potentially because of the use of computer-assisted alignment guides that lead to fewer emboli.^9,10,31,32^ Previous studies have also reported that embolization that occurs during surgical procedures is associated with both intraoperative and postoperative cardiac complications.^8,33-35^ As such, utilization of CATKAs may prevent emboli from occurring, therefore reducing emboli-related cardiac complications. In our CATKA cohort, none had a pulmonary embolism or acute myocardial infarction. Our finding that CATKAs were associated with lower odds of postoperative complications compared with conventional TKAs is further supported by a review by Hasan et al,^7^ which found that CATKAs have more favorable functional, radiological, and patient safety outcomes. Greater integration of computer navigation technology in practice may have a notable impact on the overall success of TKAs, by mitigating the odds of serious complications and improving patient outcomes.

Although our results showed that the use of computer navigation technology itself was associated with lower odds of postoperative in-hospital complications when compared with conventional TKA, CATKA was not associated with lower odds of 90-day all-cause readmissions. Other risk factors may be more influential than the technology and surgical approach when examining a 90-day episode of care that is primarily outpatient managed.^36,37^ Previous literature has shown that minority race/ethnicity, lower socioeconomic status, lower volume hospitals, and having public insurance are all associated with higher odds of 90-day all-cause readmissions.^14,18,36-38^ In the current reimbursement environment, these findings do bring into question whether hospitals—particularly those participating in bundled payment programs—may be motivated to invest in computer-assisted technology. Higher initial costs associated with CATKAs can be attributed to preoperative imaging, materials, and software. Lower in-hospital complication rates are likely to decrease hospital costs under a bundled payment model, allowing them to increase their potential profit sharing. However, to better understand whether cost savings associated with reduced in-hospital complications are sufficient to financially incentivize the adoption of computer-assisted technology despite no apparent cost savings related to 90-day readmissions, a follow-up cost analysis is warranted.

We hope that the more recent cohort-based results of this study will help increase wider adoption of CATKA. Our study corroborates a growing body of evidence that shows CATKAs are associated with lower odds of postoperative in-hospital complications, but the adoption of this technology does not reflect this.^7,8,9,10,11,12,13,31,32,33,34,35,39,40^ In the current study, CATKA represented slightly less than 10% of all TKAs. In another recent study of CATKA trends, modest increases were observed from 2010 to 2017.^14^ The use of computer-assisted navigation technology for TKAs has yet to become the primary recommendation/standard of care for unilateral uncomplicated TKA.^41^ Comparatively, increased adoption of the da Vinci Surgical System for robot-assisted prostatectomy and increased utilization of minimally invasive robot-assisted urologic surgeries were driven by greater surgical efficiency and improved patient outcomes.^42,43^ Given the better surgical and patient outcomes associated with CATKAs, the endorsement of CATKAs as a benchmark for certain patient cohorts may improve adoption.

Although this is the first *ICD-10*–based study that provides insight on in-hospital complication rates after conventional TKAs and CATKAs, it has some limitations. The study relies on a single administrative database, which likely underreports the number of CATKAs performed in the State of New York. Although coding errors are possible, studies have shown that administrative claims data are reliable for studying outcomes after joint arthroplasty.^17,18^ In addition, the data were derived from only one state. Although New York is a large, diverse state and may serve as a surrogate for national CATKA complication rates, the findings of the study are not necessarily representative of all the CATKA complication rates across the United States. Moreover, some hospital characteristics not included in our model, such as teaching status, size, and geographical location, may be associated with CATKA uptake and subsequent postoperative CATKA complication rates. For instance, large size, urban location, and nonteaching status have all been found to be associated with higher rates of CATKAs and technology-assisted KAs.^11,44^ Our analysis did not account for the possibility of selection bias, whereby the unmeasured characteristics of patients who underwent CATKA may be different from those who underwent conventional TKA. In addition, although we were able to comprehensively capture postoperative in-hospital complications, the patient-specific identifiers used in this study did not capture postdischarge complications requiring readmission if patients were readmitted into facilities outside of New York, which represents another important outcome currently captured in the Hospital Value-Based Purchasing and Comprehensive Care for Joint Replacement programs. Therefore, readmission outside of New York could be a confounding variable because patients who undergo surgery in New York may return home with complications. However, we believe that the likelihood of this happening applies similarly to patients who underwent CATKA and conventional TKA. Furthermore, the study includes data on in-hospital surgeries and does not include any surgeries performed in ambulatory surgery centers. Despite these limitations, we believe that the large sample size of the study provides valuable insight about the relationship between CATKAs and in-hospital complications across a diverse cohort.

## Conclusion

In our study cohort, patients who had CATKAs had markedly lower odds of postoperative in-hospital complications compared with those who had conventional TKAs. Our findings have important implications for increasing adoption and access to this technology.
